# Impact of obesity on outcomes of rotator cuff repair: A systematic review and meta-analysis

**DOI:** 10.1371/journal.pone.0299125

**Published:** 2024-03-13

**Authors:** Xiaojun Ma, Jia Shen, Jun Wan

**Affiliations:** 1 Department of Sports Medicine, Ningxia Hui Autonomous Region People’s Hospital, Yinchuan City, Ningxia Hui Autonomous Region, China; 2 Department of Intensive Care Unit, General Hospital of Ningxia Medical University, Yinchuan City, Ningxia Hui Autonomous Region, China; Policlinico Universitario A. Gemelli IRCCS - Universita Cattolica del Sacro Cuore Roma, ITALY

## Abstract

**Background:**

To synthesize the existing evidence on the association between obesity and rotator cuff repair outcomes such as pain, shoulder function, range of motion, and complications.

**Methods:**

We searched PubMed, EMBASE, and Scopus databases for relevant observational studies (cohort and case-control) and randomized controlled trials (RCTs). The target population in the included studies comprised adults who had undergone rotator cuff repair procedures. The outcomes of interest were functional outcomes (such as range of motion), pain scores, patient-reported outcome measures, and complication rates (such as re-repair and readmission rates). We applied random-effects models and calculated pooled effect sizes reported as standardized mean differences (SMDs) or relative risks (RRs) with 95% confidence intervals.

**Results:**

We analysed data from 11 studies. In most, the follow-up periods ranged from 12 to 60 months. Obese individuals experienced greater pain (SMD 0.30; 95% CI, 0.10, 0.50) and lower shoulder function (SMD -0.33; 95% CI, -0.54, -0.12) than other individuals in the long-term post-operative follow-up. Obese individuals also had higher risks of complications (RR 1.48; 95% CI, 1.11, 1.98) and readmission (RR 1.35; 95% CI, 1.27, 1.43), but a similar likelihood of re-repair (RR, 1.27; 95% CI, 0.82, 1.95) than non-obese/normal BMI individuals. While the forward flexion and external rotation functions were comparable, obese individuals displayed less internal rotation function than other individuals (SMD -0.59; 95% CI, -0.87, -0.30).

**Conclusion:**

Obesity was associated with unfavourable outcomes after rotator cuff surgery, including increased pain, reduced shoulder function, high risks of complications, and readmission. These findings emphasize the importance of addressing obesity-related factors to improve post-operative outcomes.

## Introduction

Obesity has emerged as a significant public health concern worldwide [1]. The impact of obesity on musculoskeletal health has become particularly evident. Recent evidence has shown that obesity could be an important risk factor for occurrence and severity of rotator cuff tears [2, 3].

These tears could lead to pain, limited range of motion, and functional impairment [4]. Rotator cuff repair (RCR) is a common orthopaedic procedure aimed at alleviating shoulder pain and restoring function in patients. Surgical repair of rotator cuff tears has become a standard approach for patients who do not respond to conservative therapies [5].

The impact of obesity on certain orthopaedic procedures, such as total hip and knee arthroplasties, has been studied; but its role in the outcome of RCR has received comparatively less attention [6–8]. However, growing evidence suggests that obesity may influence the success and complication rates of rotator cuff repair procedures. Obese individuals often experience high mechanical stresses on the shoulder joint due to their increased body weight, potentially compromising the repair’s integrity and reducing the overall surgical success [9, 10]. Moreover, obesity has been associated with a pro-inflammatory state that may hinder the healing process and increases the likelihood of postoperative complications [11, 12].

According to a recent systematic review by Marina *et al* examining the association between obesity and tendinopathy, patients with obesity faced a heightened risk of tendinopathy, as well as an increased likelihood of tendon tears and ruptures in both the upper and lower extremities [13]. Moreover, individuals with obesity also exhibited a greater risk of complications than other patients following tendon surgery [13]. That review was not specific to rotator cuff tear repair, and no comprehensive systematic review has been conducted to consolidate and analyse the collective findings on this specific topic. Therefore, we designed this comprehensive meta-analysis to pool the existing data, provide a robust evaluation of the impact of obesity on rotator cuff repair outcomes, and offer valuable insights to guide clinical decision-making.

## Methodology

### Primary objective of the review

Our primary objective was to quantitatively synthesize existing evidence from relevant studies to assess the possible associations between obesity and rotator cuff repair outcomes. We explored outcomes related to pain, shoulder function, range of motion, and complications (including risk of re-repair and readmission).

### Literature search

We conducted a comprehensive and systematic literature search on PubMed, Embase, and Scopus databases to identify all relevant studies. The search strategy included relevant keywords, synonyms, and Medical Subject Headings (MeSH) terms (S1 Table). Our search combined keywords including: (Obesity or high BMI or severely obese) AND (rotator cuff tear OR arthroscopic repair OR shoulder repair OR rotator cuff repair) AND (clinical outcomes OR pain OR functional outcome OR re-repair OR readmission OR complications). The search was limited to human studies published in English until July 15, 2023. Additionally, we manually searched the reference lists of relevant articles to supplement the electronic searches and include any potentially overlooked studies.

### Identification of eligible studies

We included studies that specifically investigated the impact of obesity on outcomes of rotator cuff repair. We considered observational investigations, such as cohort studies and case-control studies, and randomized controlled trials (RCTs). The target population comprised adult men and women who had undergone rotator cuff repair procedures. Eligible studies reported relevant outcome measures, such as re-tear rate, functional outcomes (*eg*, range of motion), pain scores, patient-reported outcome measures, and rates of complications.

We did not consider review articles, conference abstracts, or case reports. We eliminated any duplicated studies and excluded studies focusing on shoulder injuries unrelated to rotator cuff repairs, studies with insufficient data lacking necessary information for effect size calculations, or those employing inappropriate comparators. We also excluded studies exploring obesity as a risk factor for rotator cuff tear, tendonitis, or related surgical procedures.

We conducted a two-step screening process: Title and abstract screening followed by fulltext screening. Two study authors independently assessed the eligibility of each study based on the predefined inclusion and exclusion criteria. Any disagreements were resolved through discussion and, if necessary, a third author’s opinion was sought. We adhered to the PRISMA guidelines for meta-analyses [14] and registered the study protocol in the PROSPERO database (CRD42023449703).

### Data extraction and statistical analysis

Two authors independently used a standardized data extraction form to enter data. We employed the Newcastle-Ottawa Scale (NOS) to assess the studies quality [15]. The studies included had reported outcomes (particularly those related to pain and shoulder function) using different tools and scales. Therefore, for these continuous variables, we used standardized mean differences (SMDs) to generate overall pooled effect sizes. For categorical variables, we reported the pooled effect sizes as relative risks (RRs). The effect sizes were accompanied by 95% confidence intervals (CIs). To address variations in the baseline characteristics of the included studies, we applied a random-effects model for all analyses. We assessed publication bias using Egger’s test and through visual inspection of funnel plots [16]. For all analyses, we considered *P*-values lower than 0.05 as indicative of statistical significance.

## Results

**Fig 1:**
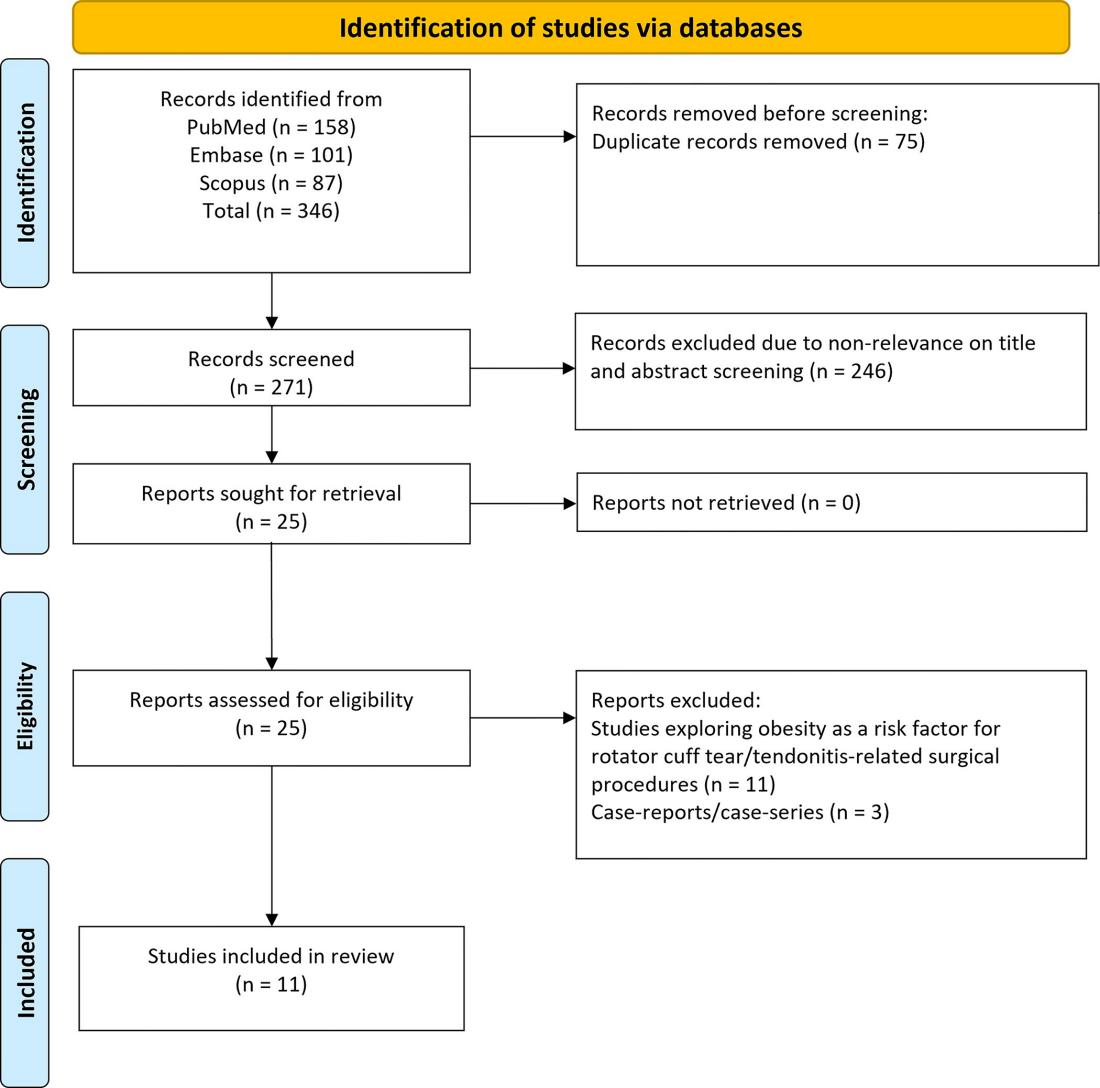
Selection process of studies included in the review.

We identified 346 studies through our search strategy. After eliminating 75 duplicates, we were left with 271 distinct studies. These were then screened on the basis of their titles and abstracts, leading to the exclusion of 246 of them. The subsequent comprehensive review of the full texts of the remaining 25 studies resulted in the exclusion of 14 more studies. Finally, we conducted our meta-analysis with data from 11 retrospective studies (Fig 1) [17–27].

Table 1 provides specific details of the studies included. Most studies were conducted in the USA (*n* = 8), the remaining studies were done in Brazil, Spain, and Germany. In eight studies, all the participants underwent arthroscopic repair of rotator cuff tears, in the other three studies, some participants underwent arthroscopic repair and some open repair. In nine studies, the follow-up period at which the outcomes were assessed was longer than 12 months (from 12 to 60 months). Four studies had NOS scores of 8 (out of the maximum attainable score of 9) and the remaining seven studies of 7 for a mean quality assessment score at 7.36, indicating acceptable quality. The details of the quality assessment are provided in S2 and S3 Tables. The total number of participants in the included studies was 331,488 out of which 45,004 were obese and 286,484 were non-obese or had normal BMIs. The studies adopted different BMI cut-offs to label obesity: Six studies used a BMI ≥30 kg/m^2^, four studies used a BMI >30 kg/m^2^, and one study used a BMI >40 kg/m^2^. The comparator groups were also heterogenous. The comparator group consisted of participants with BMIs <30 kg/m^2^ in four studies; ≤30 kg/m^2^ in three studies; between 18.5 and 24.9 kg/m^2^ in three other studies; and, <25 kg/m^2^ in one study (Table 1).

**Table 1:** Key characteristics of included studies.

Author (year of publication)	Study design	Country	Population characteristics and follow up period	Definition of obesity (comparator group)	Sample size	Newcastle Ottawa quality score
Silva (2021) [17]	Retrospective	Brazil	Mean age, 60 years; women (75%); predominantly arthroscopic repair (66%); full thickness rotator cuff tear (RCT) (85%); median follow-up of 25 months;	BMI ≥30 kg/m^2^ (non-obesity defined as BMI <30 kg/m^2^)	Obese, 13; Non-obese, 34	7
Kessler (2018) [18]	Retrospective	USA	Mean age, 58 years; men (63%); all with arthroscopic repair and full thickness rotator cuff tear (RCT); follow-up of 36 months;	BMI ≥30 kg/m^2^ (non-obesity defined as BMI <30 kg/m^2^)	Obese, 86; Non-obese, 127	7
Namdari (2010) [19]	Retrospective	USA	Mean age, 56 years; men (54%); arthroscopic repair (51%); open repair (49%); all full thickness rotator cuff tear; mean follow-up of 55 weeks (~1 year)	BMI >30 kg/m^2^ (non-obesity defined as BMI ≤30 kg/m^2^)	Obese, 57; Non-obese, 97	7
Warrender (2011) [20]	Retrospective	USA	Mean age, 66 years; men (55%); arthroscopic repair (100%); full thickness rotator cuff tear (36%); mean follow-up of 16 months	BMI >30 kg/m^2^ (non-obesity defined as BMI ≤30 kg/m^2^)	Obese, 59; Non-obese, 90	8
Kashanchi (2021) [21]	Retrospective	USA	Majority in the age group of 40–64 years (66%); men (59%); arthroscopic repair (100%); Outcomes reported at 30-day post-operative follow-up	BMI ≥30 kg/m^2^ (non-obesity defined as BMI <30 kg/m^2^)	Obese, 8973; Non-obese: 9548	7
Cruz (2023) [22]	Retrospective	Spain	Mean, 58 years; women (63%); arthroscopic repair (100%) follow-up of at least 60 months	BMI >30 kg/m^2^ (non-obesity defined as BMI ≤30 kg/m^2^)	Obese, 47; Non-obese, 33	7
Gambhir (2022) [23]	Retrospective	USA	Mean, 60 years; men (59%); arthroscopic repair (100%); mean follow-up of 28 months	BMI ≥30 kg/m^2^ (normal BMI defined as BMI <25 kg/m^2^)	Obese- 100; Normal, 86	8
Grewal (2022) [24]	Retrospective	USA	Age, older than 65 years (75%); women (51%); arthroscopic repair (100%); outcomes reported at 90-day post-operative follow-up	BMI ≥30 kg/m^2^ (non-obesity defined as BMI ≤30 kg/m^2^)	Obese, 35,723; Non-obese, 276,327	7
Fares (2022) [25]	Retrospective	USA	Mean age, 56 years; men (>50%); arthroscopic repair (100%); mean follow-up of 50 months	BMI >40 kg/m^2^ (normal BMI between 18.5 to 24.9 kg/m^2^)	Severely obese, 37; Normal, 52	8
Ateschrang (2018) [26]	Retrospective	Germany	Mean age, 59 years; arthroscopic repair (~50%); remaining mini-open repair; mean follow-up of 43 months	BMI of >30 kg/m^2^ (normal BMI between 18.5 to 24.9 kg/m^2^)	Obese, 30; Normal, 38	7
Parnes (2022) [27]	Retrospective	USA	Arthroscopic repair (100%); follow-up of 4 years	BMI of 30 to 39.9 kg/m^2^ (normal BMI between 18.5 to 24.9 kg/m^2^)	Obese, 59; Normal, 52	8

### Shoulder pain outcomes

**Fig 2:**
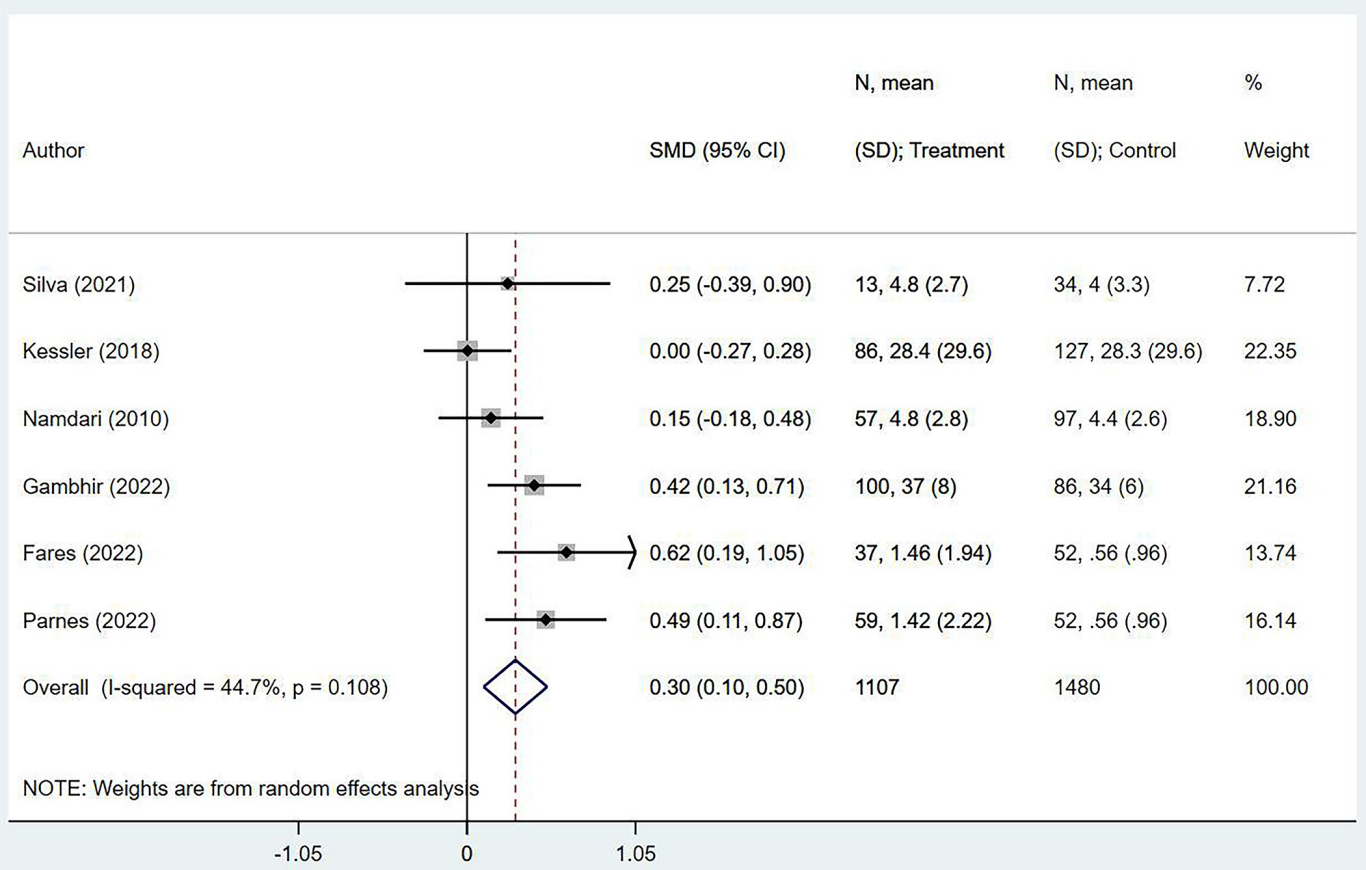
Comparison of pain scores between obese individuals and non-obese/normal BMI individuals after rotator cuff repair.

Overall, 6 studies reported follow-up pain scores. Out of these, five assessed pain using the visual analogue scale (VAS) [17–19, 25, 27] and one using the PROMIS pain interference scale [23]. In both these scales, a higher score indicates greater pain intensity [28, 29]. The pooled analysis results showed that obese participants had higher pain scores than non-obese/normal BMI participants at long term post-operative follow-ups (SMD 0.30; 95% CI, 0.10, 0.50; *n* = 6; *I*^*2*^ = 44.7%) (Fig 2).

We found no evidence of publication bias after the Egger’s test (*P* = 0.411) or upon visual inspection of the funnel plots (S1 Fig).

### Functional outcomes

**Fig 3:**
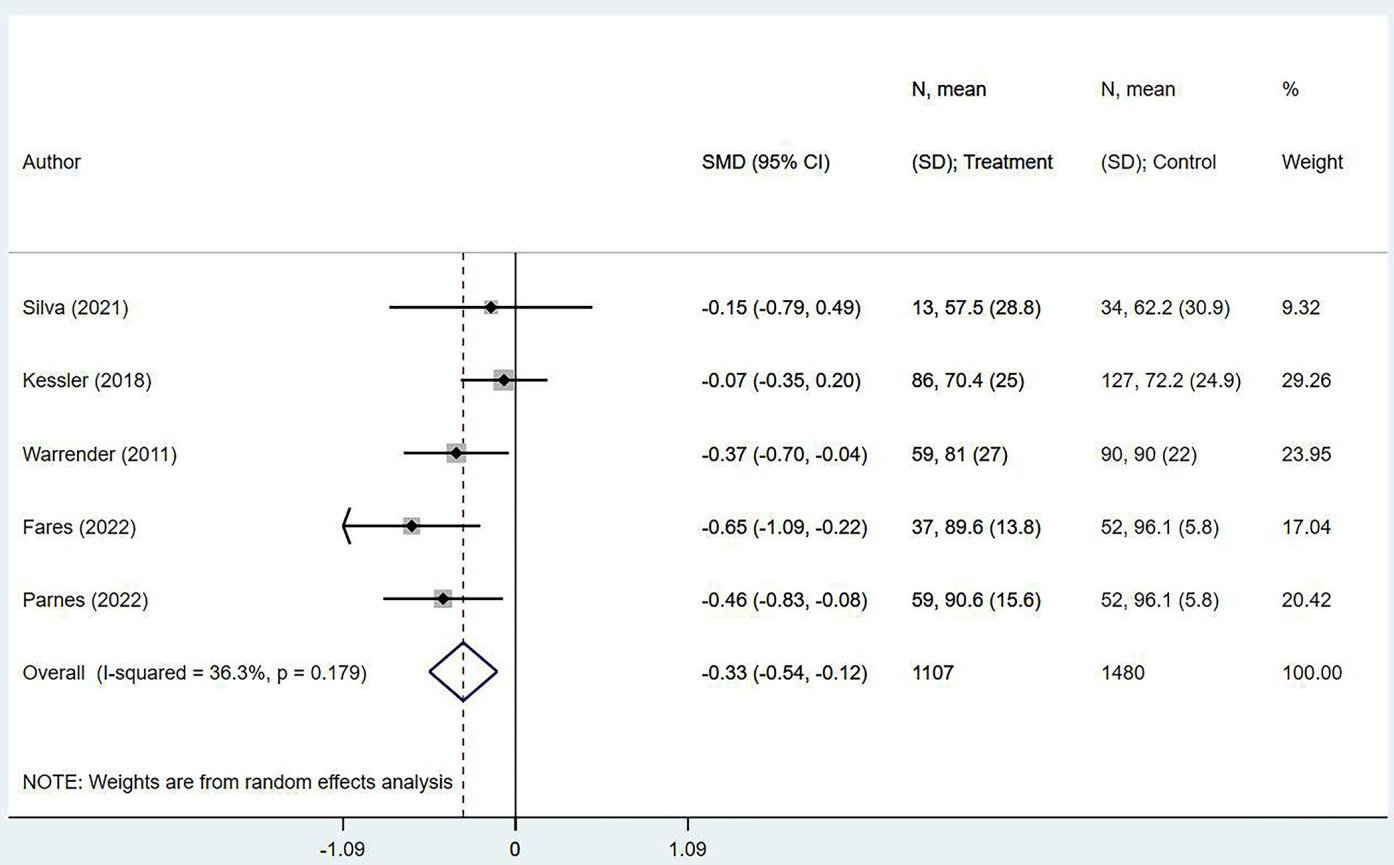
Comparison of functional outcome scores in obese and non-obese/normal BMI individuals after rotator cuff repair.

We based our pooled functional outcomes on data from five studies which used ASES scores (American Shoulder and Elbow Surgeons score), where higher scores reflect better functioning of the shoulder [30, 31]. The pooled analysis results showed that obese individuals had lower scores than non-obese/normal BMI individuals (SMD -0.33; 95% CI, -0.54, -0.12; *n =* 5; *I*^*2*^ = 36.3%) (Fig 3).

There was no evidence of publication bias on Egger’s test results (*P* = 0.490) or upon visual inspection of funnel plots (S2 Fig).

### Risks of complications

**Fig 4:**
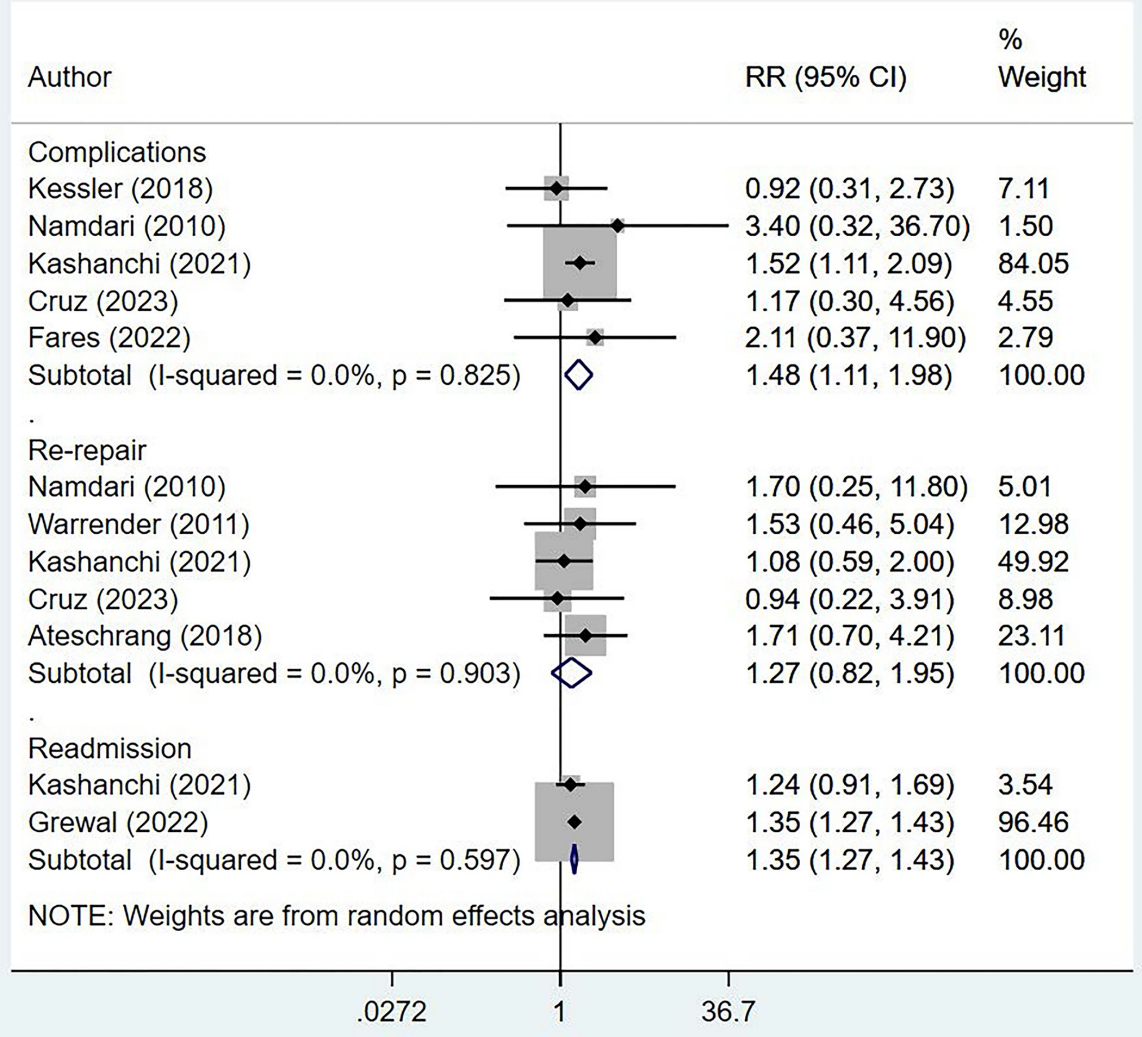
Comparison of complication risks in obese and non-obese/normal BMI individuals undergoing rotator cuff repair.

Compared with non-obese/normal BMI individuals, those with obesity had higher risks of complications (RR, 1.48; 95% CI, 1.11, 1.98; *n* = 5; *I*^*2*^ = 0.0%) and readmission (RR, 1.35; 95% CI, 1.27, 1.43; *n* = 2; *I*^*2*^ = 0.0%) (Fig 4).

The risks of need for re-repair were similar in all participant groups (RR, 1.27; 95% CI, 0.82, 1.95; *n* = 5; *I*^*2*^ = 0.0%). Commonly reported complications included urination difficulty, superficial and deep wound infections, cardiac complications, deep vein thrombosis, pulmonary and renal complications, sepsis, and non-healed rotator cuff tear. The main reasons for readmission were pneumonia, embolism, coronary atherosclerosis, infection, and chest pain. We found no evidence of publication bias on Egger’s test results (*P* = 0.671 for “any” complication and *P* = 0.504 for risk of re-repair) or upon visual inspection of the funnel plots (S3 and S4 Figs). We did a sensitivity analysis by excluding two studies (Kashanchi et al and Grewal et al) from the analysis as they had a comparatively larger sample size. We found that the risk of complications appeared to be similar between obese and non-obese individuals (RR, 1.29; 95% CI, 0.62, 2.66; n = 4; I2 = 0.0%) (S5 Fig).

### Range of motion outcomes

**Fig 5:**
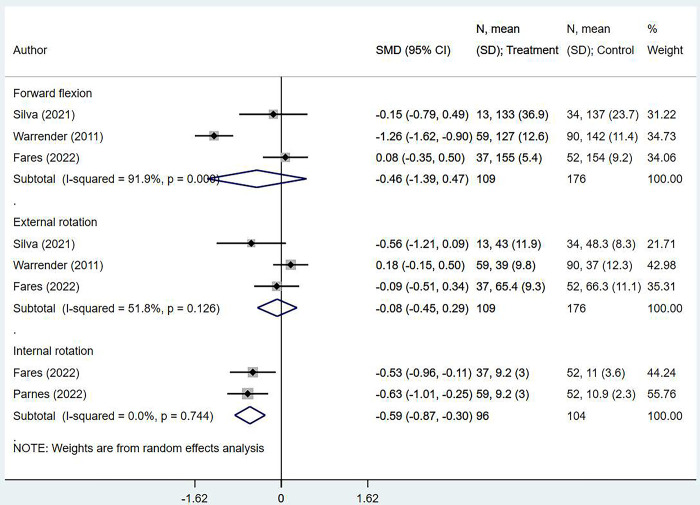
Comparison of shoulder joint ranges of motion in obese and non-obese/normal BMI individuals after rotator cuff repair.

After the tear cuff repair, all groups of individuals displayed similar degrees of forward flexion (SMD -0.46; 95% CI, -1.39, 0.47; *n* = 3; *I*^*2*^ = 91.9%) and external rotation ranges of motion (SMD -0.08; 95% CI, -0.45, 0.29; *n* = 3; *I*^*2*^ = 51.8%). However, obese individuals showed a lower degree of internal rotation than the others (SMD -0.59; 95% CI, -0.87, -0.30; *n* = 2; *I*^*2*^ = 0.0%) (Fig 5).

Egger’s test results showed no evidence of publication bias (*P*>0.05).

## Discussion

Our meta-analysis on the impact of obesity on outcomes after rotator cuff repair yielded significant and clinically relevant findings. The pooled findings suggest a clear association between obesity and unfavourable post-operative results. One of the notable findings of this meta-analysis is the more intense pain experienced by obese individuals after rotator cuff surgery. This observation has important implications for both patients and healthcare providers in the management of post-operative pain and the optimization of long-term outcomes. The association between obesity and increased post-operative pain scores may be due to different underlying mechanisms: First, excessive body weight places intense mechanical stress on the healing shoulder joint and the repaired rotator cuff tendons [9, 10]. This mechanical burden can hinder the healing process and exacerbate inflammation, resulting in heightened pain perception. Second, obese individuals often have a higher prevalence of comorbidities, such as diabetes mellitus, cardiovascular disease, and metabolic syndrome, which may influence pain perception and sensitivity [32]. Third, the chronic low-grade inflammation commonly associated with obesity may contribute to heightened pain responses, making pain management a more challenging task in this population [33]. Persistent pain can limit the willingness and ability of patients to actively participate in rehabilitation exercises and physical therapy sessions. This, in turn, may lead to a suboptimal recovery, reduced range of motion, and compromised shoulder function.

Our meta-analysis revealed a consistent and significant association between obesity and reduced shoulder function, as well as limited range of motion after rotator cuff repair. In addition to the detrimental effect on the healing process of the repaired rotator cuff tendons, excessive adipose tissue may interfere with tissue vascularity and oxygen supply, slowing down the healing process and compromising the quality of the healed tendon [34, 35]. This may result in a weaker and less functionally efficient rotator cuff, leading to reduced shoulder function and limited range of motion. Obesity is often associated with reduced muscle strength and imbalances in the musculature around the shoulder joint [36, 37]. Weakness in the rotator cuff muscles and surrounding shoulder muscles can affect joint stability and control, leading to decreased range of motion and impaired functional outcomes following rotator cuff repair. Reduced shoulder function and limited range of motion can significantly impact the activities of daily living of patients.

We found that obese patients undergoing rotator cuff repair were at risk for needing hospital readmission. But we found no evidence of a significant increase in the risk of requiring a second repair operation among obese individuals. This suggests that while obesity may be associated with a higher incidence of all complications, these complications may not necessarily lead to a higher rate of revision operations. However, it is essential to interpret this finding with caution because the risk of re-repair may be influenced by factors not fully accounted for in the included studies, such as patient compliance, surgeon experience, and variations in surgical techniques. The increased risk for complications and readmission in obese patients underscores the importance of thorough pre-operative assessments. Identifying and addressing potential risk factors associated with obesity before surgical operations can help mitigate adverse outcomes and optimize patient safety. It is worth mentioning here that addressing the statistical dilemma associated with pooling studies is crucial, particularly in interpreting findings for clinical practice. In our analysis of the risk of complications, we observed a significant dilemma linked to the inclusion or exclusion of studies with higher sample sizes. The inclusion of larger studies suggested an elevated risk of complications in obese subjects, whereas exclusion from the analysis indicated a similar risk of complications. While larger sample sizes enhance statistical power and precision, it is paramount to cautiously assess whether the observed statistical significance aligns with clinical significance. This underscores the importance of a nuanced approach when translating research findings into practical implications for clinical practice.

Our findings apply particularly in the context of the vulnerable group of elderly patients. Rotator cuff tears in the elderly are prevalent and challenging, driven primarily by age-related degenerative changes. Early diagnoses, appropriate treatments, and comprehensive rehabilitations can help alleviate pain, improve function, and enhance the quality of life for elderly individuals with rotator cuff tears. Understanding the impact of obesity on the condition is crucial for providing tailored treatment approaches that address both the tear and the underlying obesity-related factors. A multidisciplinary approach involving orthopaedic specialists, physical therapists, and nutritionists can help optimize outcomes and improve the overall well-being of these patients.

We are aware of the limitations of our meta-analysis. For the “range of motion” outcomes, there was substantial heterogeneity and there could be some potential reasons for the same. Firstly, differences in patient characteristics such as age and gender, baseline health, and preoperative conditions, may contribute to the observed heterogeneity. Additionally, variations in surgical techniques, including different approaches to rotator cuff repair and fixation methods could also be a potential contributor. Divergent postoperative rehabilitation protocols, with discrepancies in intensity, duration, and prescribed exercises, represent another potential source of variability. The duration of follow-up, ranging across studies, may impact the assessment of ranges of motion, as can the differing methods employed to measure forward flexion and external rotation. Variances in geographic locations and institutional practices can also contribute to the observed heterogeneity. Obesity is often associated with comorbidities and risk factors that can independently influence surgical outcomes. The inability to fully control for these confounding variables in the included studies may have impacted the accuracy of our observed associations. Different studies used varying BMI cut-offs to define obesity. Also, the comparator groups were different between studies. The lack of a standardized definition of obesity across all studies, variations in the control groups, and the above-mentioned inter-study differences may have introduced inconsistencies and limit the comparability of our results. Most studies were conducted in the USA and this may have limited the generalizability of the findings to other populations or contexts. Also, the studies included in our review had participants aged approximately 50–65 years. It is acknowledged that the incidence of rotator cuff tears increases with age, with higher incidence in elderly. The findings of our review could only be applicable to this relatively younger population. We emphasize the need for additional research in older populations to enhance the generalizability of our findings.

Towards the end of the discussion, we would like to mention that while our analysis suggests an elevated risk of adverse outcomes in patients with obesity, it’s crucial to recognize that there might be other influential factors that could significantly modify these outcomes. A recent investigation highlighted several variables that could impact the occurrence of retears in repairs, including tear size, the degree of fatty infiltration in rotator cuff muscles, patient age, and the use of a double-row construct [38]. Moreover, this study suggested that patient-reported outcomes may be influenced by factors like the percentage of nonsmokers, the proportion of women in the study, and the timing of initiating active range of motion and strengthening exercises. However, the extent to which these factors independently contribute to outcomes remains unclear, as the interplay between them wasn’t explicitly determined in the analysis. Similarly, discerning whether obesity directly causes poor outcomes or whether there are associated factors, presents a complex challenge that may not be easily disentangled, and future studies should be directed to provide insights into the same.

## Conclusion

The findings of this meta-analysis provide evidence that obesity is associated with adverse outcomes after rotator cuff repair. Healthcare providers should be aware of the impact of obesity during pre-operative evaluation and emphasize weight management strategies to optimize surgical outcomes for obese patients. Further research and prospective studies are needed to better understand the underlying mechanisms and to develop targeted interventions to improve post-operative results in this patient population.

## Supporting information

S1 Checklist PRISMA 2020 checklist. (DOCX)

S1 Fig Funnel plot for pain scores. (TIF)

S2 Fig Funnel plot for functional outcome scores. (TIF)

S3 Fig Funnel plot for risk of any complication. (TIF)

S4 Fig Funnel plot for risk of re-repair. (TIF)

S5 Fig Sensitivity analysis for risk of complications and re-repair. (TIF)

S1 Table Search strategy used to identify studies. (DOCX)

S2 Table Author’s judgements about study quality using the adapted Ottawa-Newcastle risk of bias assessment tool. (DOCX)

S3 Table Author’s judgements about study quality using the adapted Ottawa-Newcastle risk of bias assessment tool. (DOCX)
