# Supplementary figures and images for: Impact of obesity on outcomes of rotator cuff repair: A systematic review and meta-analysis
Source: PLoS One. 2024 Mar 13;19(3):e0299125. doi: 10.1371/journal.pone.0299125 (PMC10936781; doi:10.1371/journal.pone.0299125)

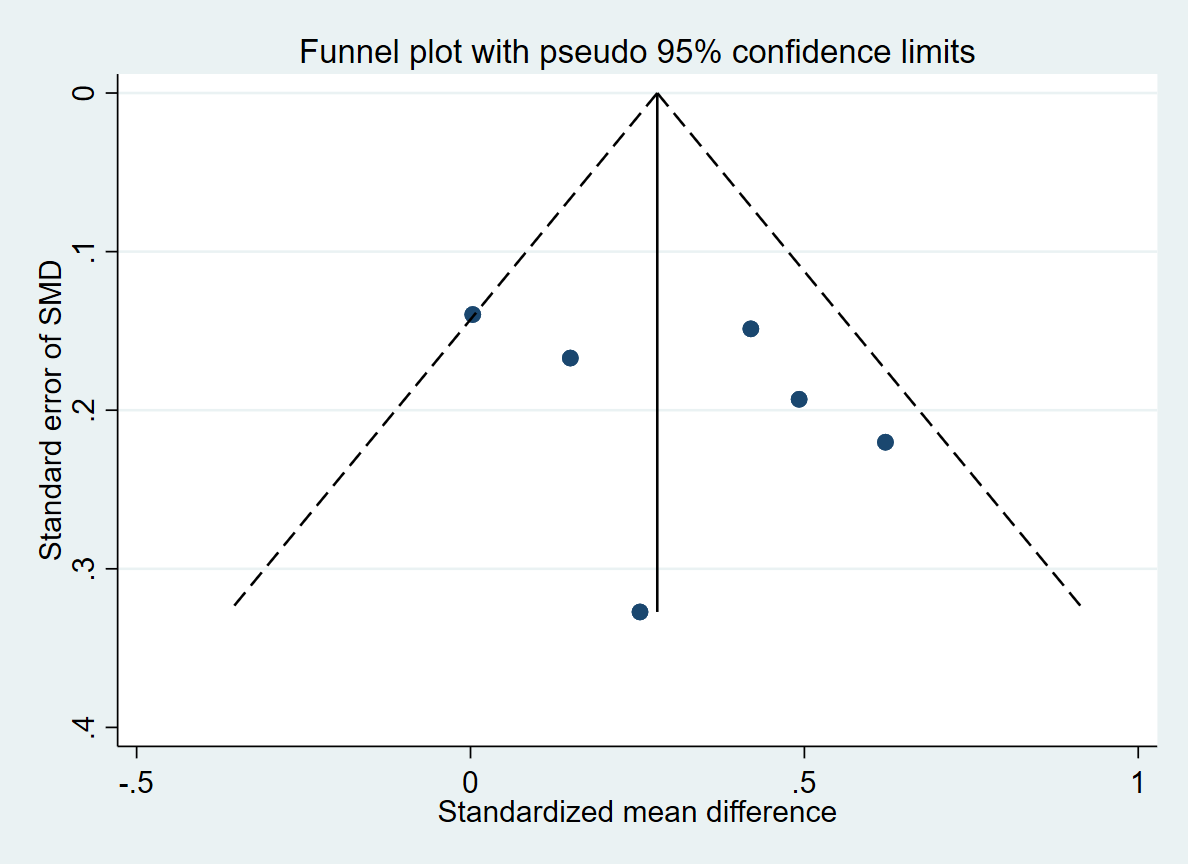

Supplement: S1 Fig — (TIF) [file pone.0299125.s002.tif]

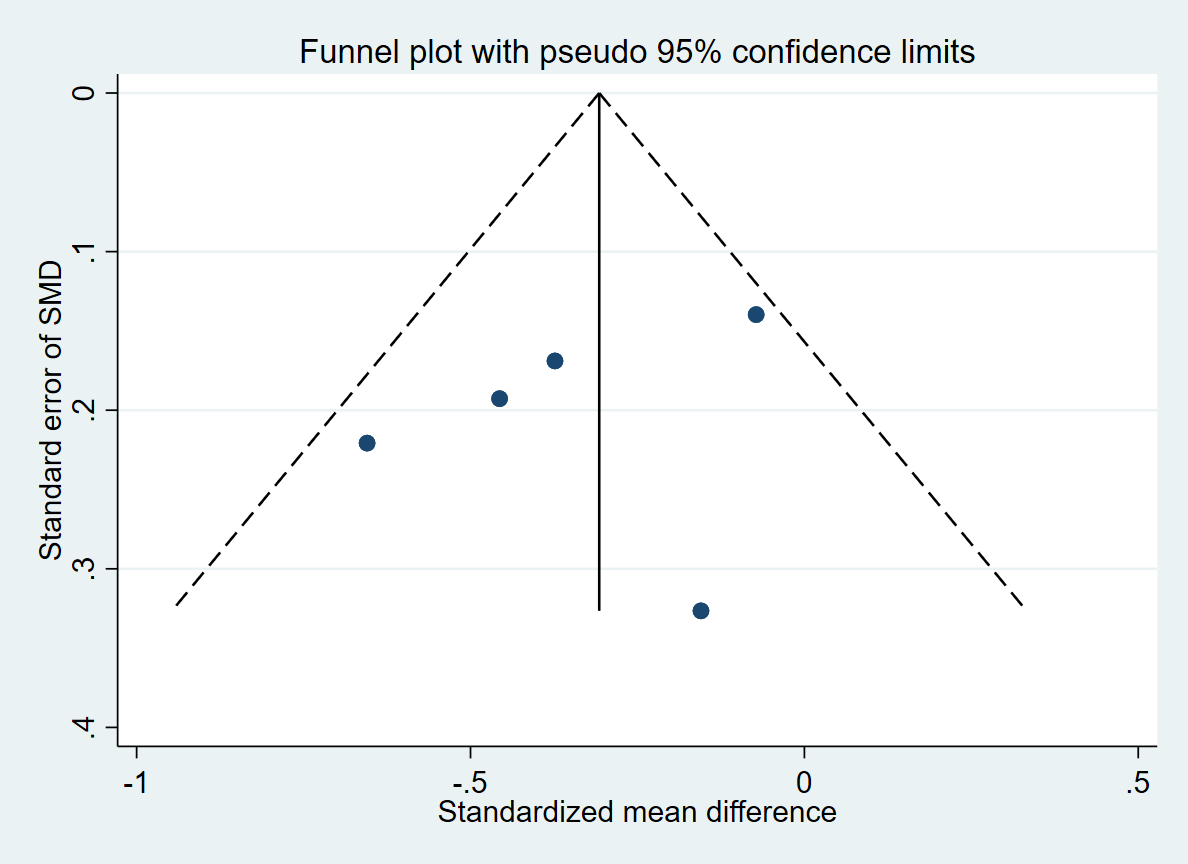

Supplement: S2 Fig — (TIF) [file pone.0299125.s003.tif]

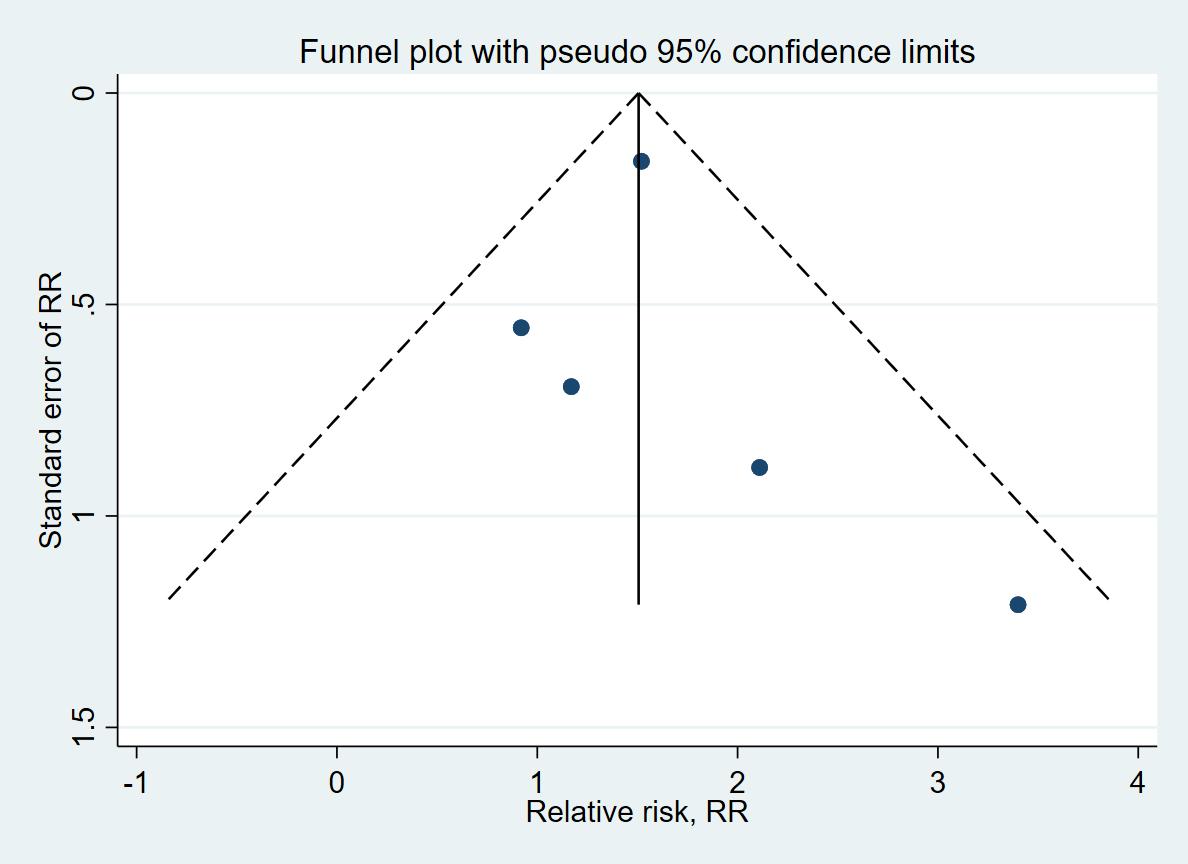

Supplement: S3 Fig — (TIF) [file pone.0299125.s004.tif]

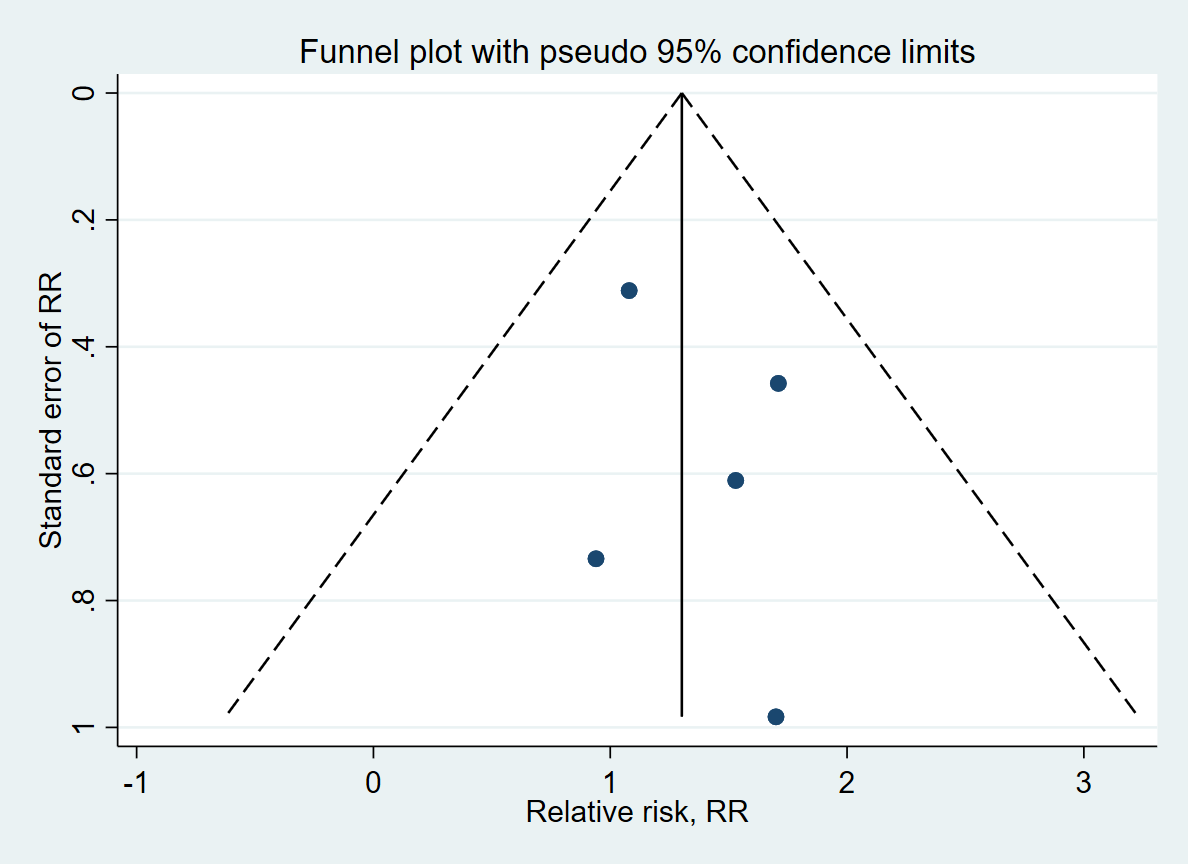

Supplement: S4 Fig — (TIF) [file pone.0299125.s005.tif]

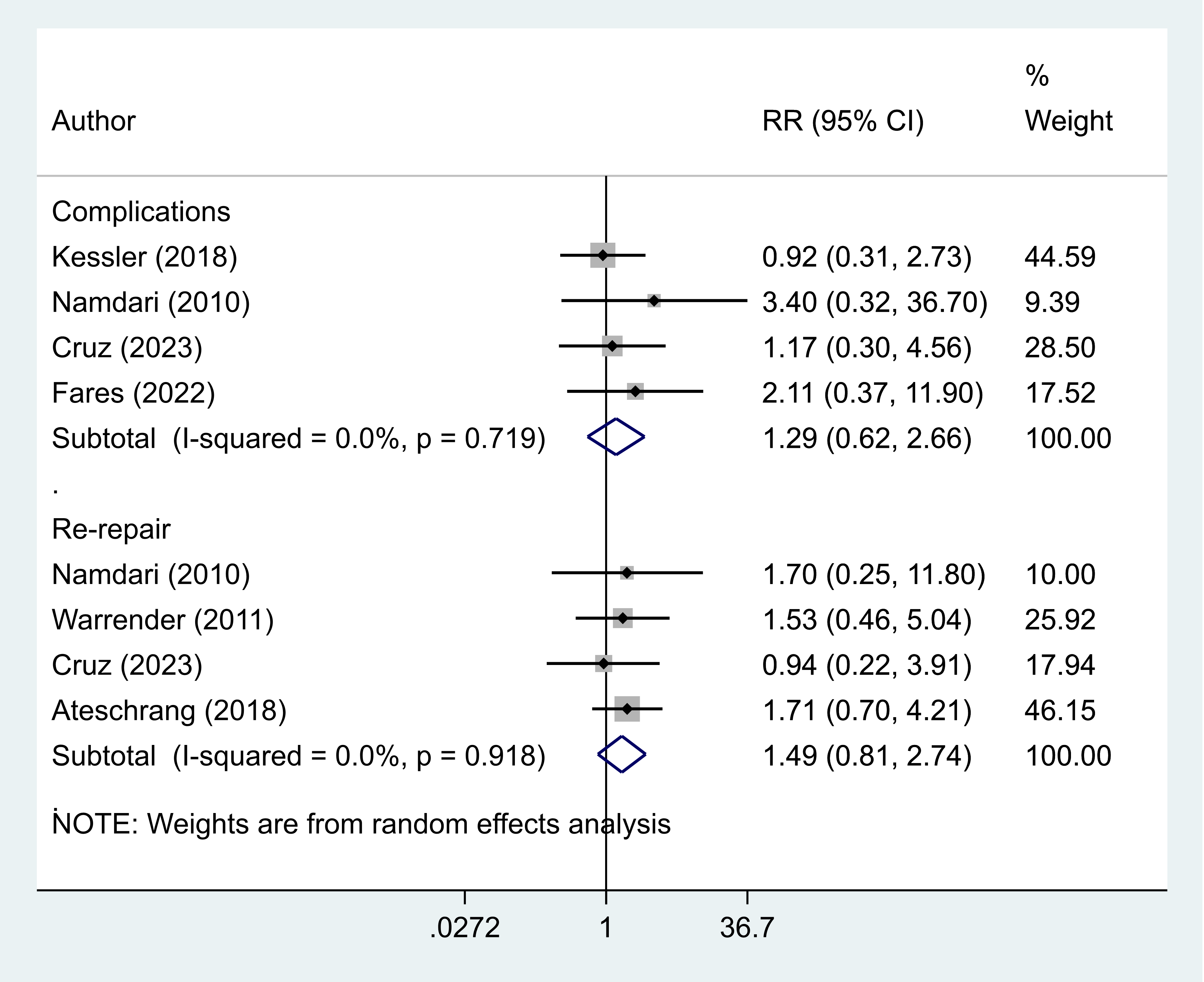

Supplement: S5 Fig — (TIF) [file pone.0299125.s006.tif]
