# Supplementary material for: Impact of obesity on outcomes of rotator cuff repair: A systematic review and meta-analysis
Source: PLoS One. 2024 Mar 13;19(3):e0299125. doi: 10.1371/journal.pone.0299125 (PMC10936781; doi:10.1371/journal.pone.0299125)
Supplement: S1 Table — (DOCX) [file pone.0299125.s007.docx]

**S1 Table. Search strategy used to identify studies**

**Search strategy for PubMed**

("obesity" OR "obese" OR “severely obese” OR "body mass index" OR "BMI" OR “High BMI” OR "adiposity") AND

(“rotator cuff tear” OR "rotator cuff repair" OR "rotator cuff surgery" OR "rotator cuff reconstruction" OR “arthroscopic repair” OR “shoulder repair” ) AND

("clinical outcomes" OR “pain” OR "complications" OR "success" OR "recovery" OR "functional outcomes" OR “re-repair” OR “readmission”) AND

("humans"[MeSH Terms]) AND ("English"[Language])

**Search strategy for Embase**

('obesity' OR 'obese' OR 'body mass index' OR 'BMI' OR 'adiposity') AND

('rotator cuff repair' OR 'rotator cuff surgery' OR 'rotator cuff reconstruction') AND

('outcomes' OR 'complications' OR 'success' OR 'recovery' OR 'functional outcomes') AND

('human'/exp) AND ('English'/exp)

**Search strategy for Scopus**

TITLE-ABS-KEY ('obesity' OR 'obese' OR 'body mass index' OR 'BMI' OR 'adiposity') AND

TITLE-ABS-KEY ('rotator cuff repair' OR 'rotator cuff surgery' OR 'rotator cuff reconstruction') AND

TITLE-ABS-KEY ('outcomes' OR 'complications' OR 'success' OR 'recovery' OR 'functional outcomes') AND

INDEXTERMS ('human') AND DOCUMENTTYPES (ar)
