# Supplementary material for: Impact of obesity on outcomes of rotator cuff repair: A systematic review and meta-analysis
Source: PLoS One. 2024 Mar 13;19(3):e0299125. doi: 10.1371/journal.pone.0299125 (PMC10936781; doi:10.1371/journal.pone.0299125)
Supplement: S2 Table — (DOCX) [file pone.0299125.s008.docx]

**S2 Table. Author’s judgements about study quality using the adapted Ottawa-Newcastle Risk of Bias Assessment tool**

|  | Silva (2021) | Kessler (2018) | Namdari (2010) | Warrender (2011) | Kashanchi (2021) | Cruz (2023) |
| --- | --- | --- | --- | --- | --- | --- |
| Representativeness/appropriateness of participant selection  Random or consecutive recruitment=Y  Convenience sample=N  Not reported or unclear | Y | Y | Y | Y | Y | Y |
| Control for baseline differences in cohorts  Similarity of groups at baseline or adjustment in analyses=Y  No attempt to control or adjust=N  Not reported=NR | Y | N | N | Y | N | Y |
| Loss to follow-up  Explanation provided for loss of participants and/or intention to treat=Y  No explanation =N | Y | Y | N | Y | Y | N |
| Masking of exposure to outcomes assessor  Description of masking=Y  No masking or no description =N | N | Y | Y | Y | Y | N |
| Ascertainment of condition  Description of ascertainment/diagnostic criteria=Y  No description or patient self-report=N | Y | Y | Y | Y | Y | Y |
| Documentation of other treatment modalities  Documentation=Y  No documentation=N | Y | N | Y | Y | N | Y |
| Extent to which valid outcomes are described  Adequate description of outcome=Y  Insufficient detail regarding outcome or follow-up time=N | Y | Y | Y | Y | Y | Y |
| Prespecification of harms, mode of harms collection  Description of a list of harms assessed or monitoring=Y  No such description or passive harms collection=N  No adverse events reported=NA | N | Y | Y | N | Y | Y |
| Financial Conflict of interest (COI)  Funding source reported=Y  Funding source not reported=N | Y | Y | Y | Y | Y | Y |
